# Exploration of Optical Fiber and Laser Cutting Head Applications in High-Radiation Environments for Fast Reactor Spent Fuel Reprocessing

**DOI:** 10.3390/s25010031

**Published:** 2024-12-24

**Authors:** Qi Chen, Jiarong Zheng, Jia Zhou, Zhengbin Chen, Zengliang Mo, Zhi Cao, Chunwei Tang, Tianchi Li, Fang Liu, Taihong Yan, Weifang Zheng

**Affiliations:** 1 China Institute of Atomic Energy, P.O. Box 275 (26), Beijing 102413, Chinaliufang40131@163.com (F.L.);; 2 School of Mechanical Science and Engineering, Huazhong University of Science & Technology, Wuhan 430074, China; 3 GZ Photonics Technology Co., Ltd., Dongguan 523808, China

**Keywords:** gamma irradiation, laser cutting, fast reactor assembly, surface roughness

## Abstract

Fast-neutron reactors are an important representative of Generation IV nuclear reactors, and due to the unique structure and material properties of fast reactor fuel, traditional mechanical cutting methods are not applicable. In contrast, laser cutting has emerged as an ideal alternative. However, ensuring the stability of optical fibers and laser cutting heads under high radiation doses, as well as maintaining cutting quality after irradiation, remains a significant technical challenge. Here, we study the performance changes in optical fibers exposed to a total radiation dose of 10^5^ Gy, focusing on power transmission and thermal characteristics. By integrating irradiated optical fibers with irradiated laser cutting heads, simulated cutting experiments on the hexagonal tubes of spent fuel from fast reactors (fast reactor simulation assembly) were conducted. Critical cutting quality parameters, including kerf width, surface roughness, and slagging length, were analyzed. The results indicate that, while the power transmission performance of irradiated optical fibers shows slight degradation, its impact on cutting quality is minimal. High-quality cutting can still be achieved under optimized parameters. This study confirms the feasibility of laser cutting technology in high-radiation environments and provides essential technical support for its application in nuclear fuel reprocessing.

## 1. Introduction

At present, thermal reactors, which primarily rely on ^235^U [1], dominate nuclear energy production. However, ^235^U constitutes only 0.7% of natural uranium, while ^238^U, which accounts for 99.3%, cannot undergo fission in thermal neutron reactors. The limited availability of uranium resources raises concerns about the potential depletion of ^235^U. To address this issue, fast-neutron breeder reactors have emerged [2,3] as representatives of fourth-generation advanced nuclear reactors [4,5]. These reactors significantly enhance the utilization of natural uranium, increasing efficiency to over 60%, while also reducing nuclear waste and pollutant emissions, thereby contributing to sustainable and environmentally friendly development.

Unlike thermal neutron reactors, fast reactor fuel presents unique challenges for reprocessing due to its specialized structural composition and higher burnup levels [2,6]. Nowadays, the reprocessing of nuclear fuel from fast-neutron reactors is a key area of technological research [1,7,8,9,10,11]. Traditional reprocessing methods typically begin by shearing the fuel rods, followed by dissolving them in nitric acid [12,13,14]. The resulting solution is then filtered to remove undissolved particles and cladding debris [15,16]. Subsequently, the acidity of the solution is adjusted to optimize the valence states of uranium and plutonium to suit the requirements of the subsequent solvent extraction process. The primary distinction and challenge in reprocessing fast reactor fuel, compared to traditional pressurized water reactor (PWR) fuel, lies in the headend treatment stage of the process [17,18,19]. Mechanical shearing, widely employed for PWR spent fuel, is not suitable for fast reactor assemblies due to the higher hardness and ductility of stainless-steel hexagonal tubes [20,21]. Using a bundled shearing approach can flatten the ends of fuel segments, leading to slower dissolution rates or incomplete dissolution, which in turn negatively impacts subsequent processing steps [22]. To address these issues, alternative shearing methods are necessary for the efficient reprocessing of fast reactor spent fuel.

A laser dismantling and cutting system has been developed specifically for fast reactor spent fuel [23,24], as it has been shown that laser technology offers significant advantages in component cutting due to its precision and efficiency [25,26,27]. Laser cutting offers numerous advantages, including faster cutting speeds, reduced assembly complexity, and deformation-free cutting surfaces without residual stress [28,29]. This technology has already been adopted in the nuclear industry by several countries [29,30,31,32]. The laser dismantling system is designed to remove the hexagonal tube and end structures of the fuel assembly while preserving the integrity of the fuel rods [33], which are then cut into shorter segments. A key step in dismantling a fast reactor assembly is the circumferential cutting of the hexagonal tube [2]. This process requires high precision to ensure the hexagonal tube is cleanly severed while minimizing any potential damage to the internal fuel rods [34]. As a result, laser cutting technology must meet stringent technical requirements to perform this operation effectively and reliably [35,36,37].

The key challenge in applying laser technology to the headend treatment stage of nuclear fuel reprocessing lies in performing the whole cutting process within a hot cell, which is completely isolated from the external environment [32]. In the hot cell, solid and gaseous media are disconnected, and the system operates under high-radiation conditions. Spent fast reactor fuel exhibits extremely high levels of radioactivity, with surface gamma dose rates reaching 10^3^ Gy/h or higher. Before integrating laser technology into the dismantling and shearing processes of fast reactor nuclear fuel reprocessing, it is crucial to investigate the performance changes and radiation resistance of laser cutting heads and optical fibers under high-dose irradiation environments. It is also important to assess the quality of the cut in extreme radiation environments [38]. These evaluations help advance the use of laser technology in nuclear fuel reprocessing. High-radiation conditions, including X-rays, β rays, γ rays, and neutron radiation, can cause radiation damage to the optical components of the fibers and the laser cutting heads, leading to a reduction in the signal transmission capability and comprehensive performance degradation. In severe cases, such damage can compromise the safety and reliability of cutting operations. Gamma rays, in particular, which are high-energy electromagnetic waves with shorter wave lengths and higher frequencies than alpha and beta rays, are characterized by greater penetration power causing more significant damage to solid materials [39]. As a result, studies on the irradiation performance of optical fibers primarily focus on their behavior under γ radiation.

This study evaluates the feasibility of laser energy transmission under high-radiation conditions by examining the appearance of irradiated optical fibers through microscopic observation, along with analyzing output power variations and temperature changes. Additionally, the irradiated optical fiber was integrated with an irradiated laser cutting head to conduct a comprehensive assessment of the cutting quality for a fast reactor simulation assembly hexagonal tube. The results confirm the feasibility of applying laser shearing technology under a total γ-irradiation dose of 10^5^ Gy. This research not only advances the development of laser technology in the field of nuclear fuel reprocessing but also establishes a strong foundation for the practical implementation of laser shearing systems in industrial applications.

## 2. Experimental Methods and Materials

### 2.1. Experimental Subjects

**Figure 1:**
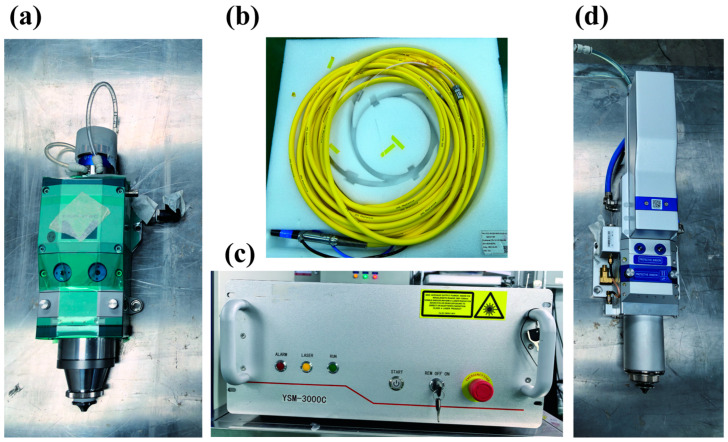
Experimental equipment: (**a**) gamma-irradiated laser cutting head, (**b**) energy transmission optical fiber, (**c**) laser source, (**d**) non-irradiated laser cutting head.

The optical fiber used in this experiment was supplied by IPG Photonics (Marlborough, MA, USA), model FF HLC-8-100/200-20 (Figure 1c). The γ-irradiated laser cutting head was also sourced from IPG Photonics (USA), model ProCutter 100/150 Q_C2 (Figure 1a). For comparison, an unirradiated laser cutting head from Han’s Laser, model HC-15 (Figure 1d), was employed. The irradiation was performed using a Co-60 gamma source, with the laser cutting head and optical fiber exposed to a radiation dose rate of 2000 Gy/h and a total accumulated dose of 10^5^ Gy.

**Figure 2:**
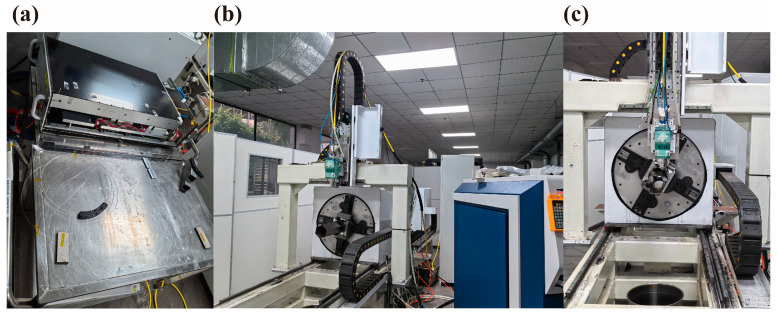
The laser dismantling and cutting system: (**a**) the laser source connected to the irradiated optical fiber, (**b**) a side view of the rotary cutting platform, (**c**) the front view of the rotary cutting platform.

The laser used for cutting was a YSM-3000C, a 3000 W laser customized by Guozhi Laser Company (Wuhan, China) (Figure 1b). The optical fiber to be studied was connected to this laser (Figure 2a). The setup also included key components such as a water cooling system, a gas cylinder assembly, and a control cabinet. The cutting experiments for the fast reactor assembly were conducted on a rotary cutting platform measuring approximately 3 m in length and 2 m in height (Figure 2b,c). This platform is designed to facilitate the rotation and cutting of a stainless-steel assembly with precision.

### 2.2. Analytical Methods

**Figure 3:**
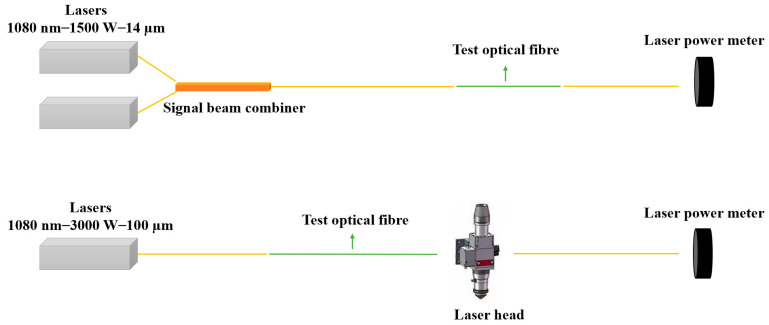
Schematic of the power testing method.

The experiment utilized a Thorlabs Vytran (Newton, NJ, USA) LDC401 large-core optical fiber cleaver and a Fujikura (Tokyo, Japan) FSM-100M optical fiber fusion splicer. Infrared thermal imaging was conducted using a FLIR-T530 (Wilsonville, OR, USA), and output power measurements were performed with a Gentec-EO laser power meter (Quebec City, QC, Canada). Output power was measured both directly from the fiber connected to the power meter probe, as well as from the laser cutting head after the fiber was connected to the laser and cutting head (a schematic of the power testing method is shown in Figure 3). Temperature measurements were also conducted simultaneously during the output power tests to monitor thermal variations.

**Figure 4:**
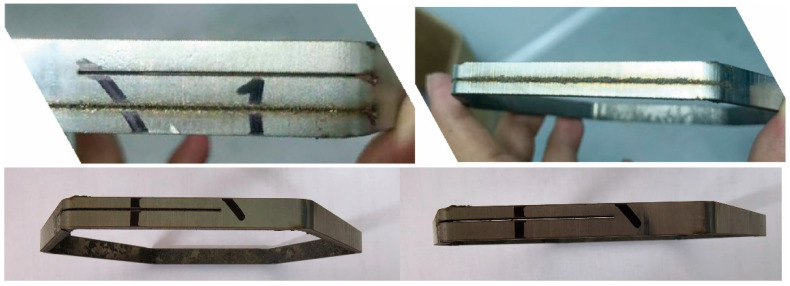
Sample of laser cut hexagonal tube.

During the cutting of fast reactor assembly hexagonal tubes, each sample underwent two cuts with identical parameters. The first cut was a semicircular cut, used to measure the joint seam, while the second cut was a full circular cut, aimed at surface characterization, including roughness measurement and slag residue observation under an optical microscope. (Figure 4) All cutting experiments were performed in an air-blown atmosphere with a gas pressure of 12 MPa and a focal position set to −2.5 mm.

The cutting quality analysis involved the following steps: (1) Photographing and visually inspecting the internal rod components and winding wires. (2) Using an optical microscope to capture images for measuring the cut width and slag residue length. (3) Measuring surface roughness with a surface roughness tester.

The brand and model of the optical microscope: Wan Hao Imaging, VMS-4030. The brand and model of the surface roughness gauge: Wenzhou Jingcheng Measurement Equipment Co., Ltd., Wenzhou, China, TR100 (test duration: 10 s; sampling length: 2.5 mm). 

## 3. Results and Discussion

### 3.1. Microscopic Characterization of Optical Fibers

**Figure 5:**
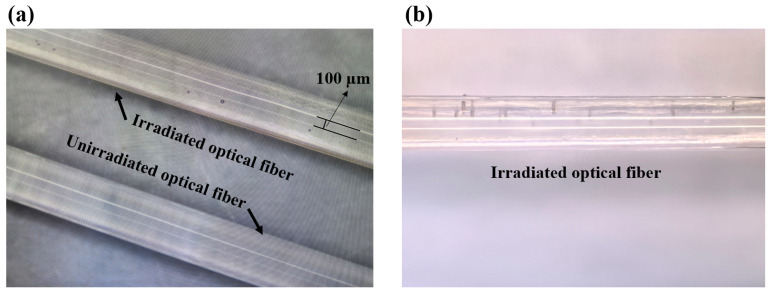
Optical fiber microscope images: (**a**) yellowish surface and (**b**) irradiation-induced cracks.

The appearance of optical fibers before and after γ-irradiation was compared using an optical microscope (Figure 5a,b). The unirradiated fiber maintained a colorless, transparent appearance. While the fiber exposed to high-dose γ-irradiation exhibited significant changes, including a yellowish surface and noticeable cracks. Some cracks appeared black, suggesting potential burn marks. These findings indicate that high-dose γ-irradiation induces substantial radiation damage to the optical fiber material.

The effects of γ-irradiation on the optical fiber are primarily manifested in the following aspects: (1) Color change—the fiber changes from colorless and transparent to yellowish, indicating that irradiation induces chemical modifications or structural reorganization within the material, potentially associated with radiation-induced optical defects. (2) Surface cracks—prominent cracks were observed on the surface of the irradiated fiber, reflecting the mechanical degradation of the coating material or cracking due to thermal stress. The blackened areas of the cracks likely result from localized burning or the deposition of decomposition byproducts caused by high-energy radiation. (3) Material damage—these observations demonstrate significant damage to both the coating and core materials of the fiber, including discoloration, surface cracking, and potential mechanical performance degradation. In summary, high-dose γ-irradiation profoundly impacts the physical and chemical properties of optical fibers, highlighting the limited resistance of the material under irradiation environments.

### 3.2. Output Power Measurement

**Figure 6:**
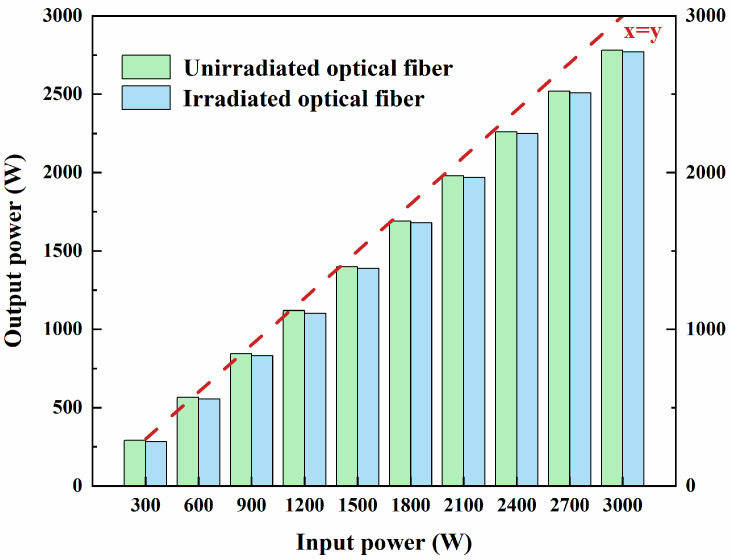
Relationship between the input and output power of the optical fiber.

To assess the energy transmission performance of irradiated optical fibers, the experiment measured the output power of the standalone fiber as well as the output power with the fiber connected to the laser cutting head. Figure 6 illustrates the output power variations in irradiated and unirradiated fibers under different input power conditions. Across all tested input power levels, the output power of the unirradiated fiber was consistently slightly higher than that of the irradiated fiber. For instance, at the maximum input power of 3000 W, the unirradiated fiber achieved an output power of 2780 W, while the irradiated fiber delivered 2770 W, a reduction of only about 0.36%. This minor power attenuation suggests that high-dose γ-irradiation has a negligible impact on the fiber’s energy transmission performance. Although the irradiated fiber exhibits a slightly lower output power, the difference is minimal and is unlikely to significantly affect laser transmission or cutting performance in practical applications.

**Figure 7:**
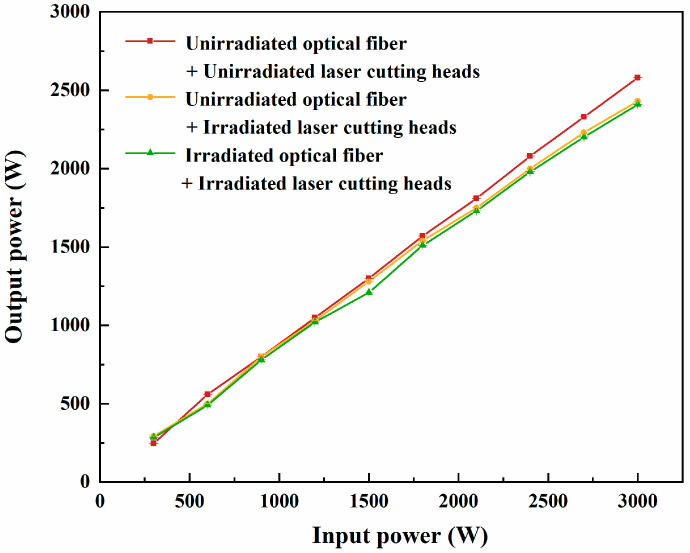
Relationship between the input and output power of the optical fiber connected to the laser cutting head.

Figure 7 illustrates the relationship between the input power and output power of the optical fibers connected to laser cutting heads, comparing three configurations: (1) an unirradiated fiber connected to an unirradiated laser cutting head, (2) an unirradiated fiber connected to an irradiated laser cutting head, and (3) an irradiated fiber connected to an irradiated laser cutting head. The output power of the unirradiated fiber connected to the irradiated cutting head was slightly lower than that of the unirradiated fiber connected to the unirradiated cutting head, with only minor variations observed. At higher input power levels, the output power of the irradiated fiber connected to the irradiated cutting head showed a reduction of less than 3% compared to the unirradiated fiber connected to the unirradiated cutting head. This indicates that the system maintains high transmission efficiency even under irradiation conditions. High-dose γ-irradiation affects the performance of the optical fiber and laser cutting head in the form of a slight reduction in output power, but the attenuation is minimal. The performance of the laser system remains fully capable of meeting the operational requirements for cutting fast stack components in practical scenarios.

### 3.3. Optical Fiber Temperature Rise Experiment

**Figure 8:**
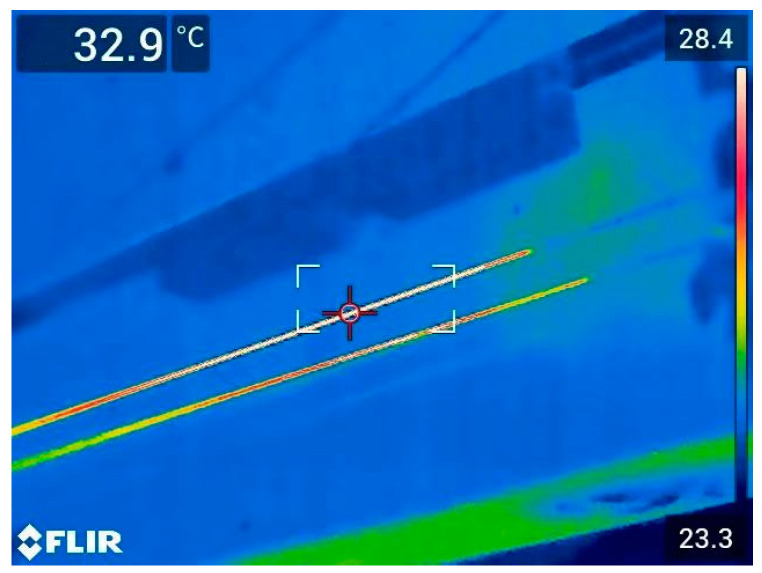
Temperature variation in an irradiated optical fiber monitored by infrared thermal imaging without the laser cutting head connected.

When deploying optical fibers in high-dose irradiation environments, it is crucial to evaluate changes in their energy transmission performance, with a particular focus on temperature variations during transmission. Figure 8 presents temperature changes in the irradiated optical fiber during power transmission, as monitored by an infrared thermal imager, when not connected to a laser cutting head. Prolonged exposure to elevated temperatures can result in fiber melting, deformation, or even failure. Such conditions may also compromise the normal operation of the optical fiber system and the laser source, potentially creating safety risks.

**Figure 9:**
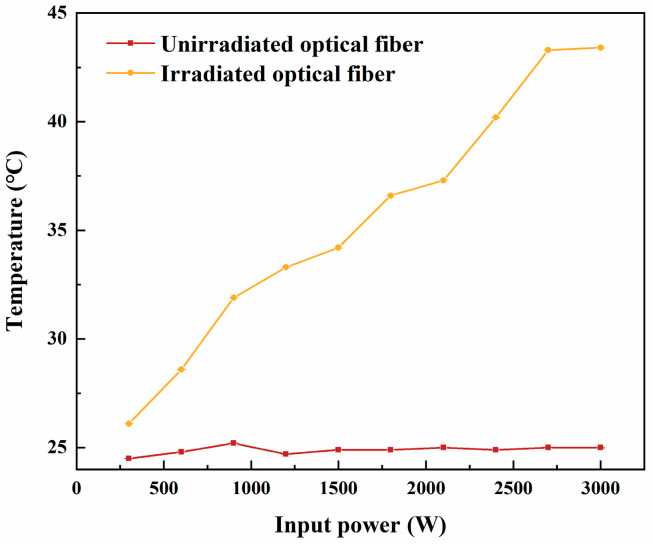
Temperature variation in an irradiated optical fiber with input power, measured without the laser cutting head connected.

Figure 9 shows the temperature variation in the irradiated optical fiber as a function of input power when not connected to a laser cutting head. For the unirradiated fiber, the surface temperature remained stable across the entire power range, with only a slight variation between 24.5 °C and 25.2 °C. This indicates that the unirradiated fiber exhibits excellent thermal stability during power transmission, with negligible thermal effects, regardless of input power or transmission duration. In contrast, the surface temperature of the irradiated fiber increased significantly with higher input power. At an input power of 3000 W, the surface temperature of the irradiated fiber reached approximately 45 °C. This increase is likely due to changes in the thermal properties of the fiber’s coating or internal structure caused by irradiation, such as reduced thermal conductivity or the accumulation of radiation-induced defects that hinder heat dissipation. The temperature monitoring results indicate that while irradiated fibers are affected by thermal effects during high-power laser transmission, they remain functional for actual cutting within the tested power range and irradiation dose. However, further increases in input power, dose rate, or cumulative dose are expected to make temperature a critical limiting factor, potentially affecting cutting performance.

**Figure 10:**
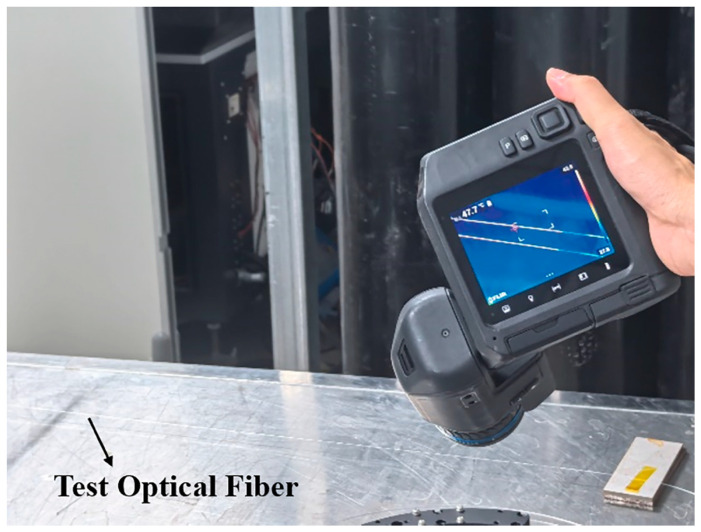
Temperature variation detected during the actual cutting process (with the laser cutting head connected).

Figure 10 illustrates the temperature variations observed during the actual cutting process with the laser cutting head connected. In this experiment, the irradiated optical fiber was coupled with the irradiated laser cutting head to simulate the cutting conditions of fast reactor assembly hexagonal tubes. Real-time temperature monitoring was conducted using infrared thermal imaging. The surface temperatures of the optical fiber varied depending on the cutting parameters and output power settings. Fortunately, under all tested conditions, the temperatures remained within a relatively low range. The maximum observed temperature was 48 °C, which did not exceed the safe operating thresholds of either the optical fiber or the cutting head. Under a cumulative irradiation dose of 10^5^ Gy, neither the irradiated optical fiber nor the irradiated laser cutting head exhibited overheating or significant damage during the cutting process. This indicates that the thermal stability and energy transmission capabilities of the irradiated optical fiber and laser cutting head remain sufficient for practical use, demonstrating their potential for extended operation in such high-radiation environments.

### 3.4. Laser Cutting Process Quality

The hexagonal tubes of the fast reactor assembly were first cut by connecting an unirradiated optical fiber to an unirradiated laser cutting head (Experiment Group 1). Drawing from prior experimental experience, the optimal cutting parameters were determined. Previous studies revealed that while faster cutting speeds can enhance efficiency, they significantly compromise cutting quality. Therefore, a consistent cutting speed of 4.5 m/min was used for this group of experiments. Subsequently, using the optimized parameters from Experiment Group 1, the irradiated optical fiber was connected to the irradiated laser cutting head for another cutting test on a hexagonal tube of the fast reactor assembly (Experiment Group 2). Parameters were adjusted to identify the optimal conditions, and the cutting quality between the two experimental groups was compared. Table 1 outlines the specific cutting parameters for each experiment, including cutting speed and power. Power adjustment proved critical in determining cutting performance, as insufficient power led to incomplete cutting, while excessive power caused increased thermal damage to the material. To further compare the cutting performance of the two experimental groups and assess the cutting stability of Experiment Group 2, additional cutting tests were conducted at the laser’s maximum power (3000 W).

**Figure 11:**
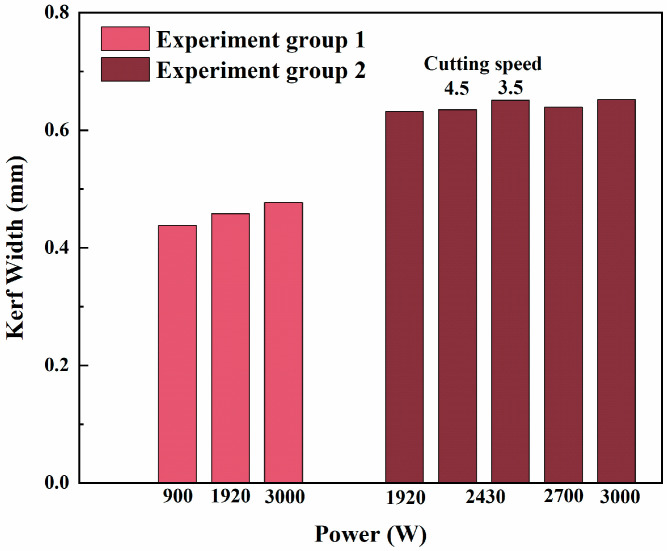
Kerf width under different cutting parameters.

Figure 11 illustrates the kerf widths obtained under different laser cutting parameters, comparing the results of the unirradiated group (Experiment Group 1) and the irradiated group (Experiment Group 2). Based on the data analysis and experimental comparison, the following conclusions can be drawn: In Experiments 1 and 5, the laser failed to completely penetrate the hexagonal tube due to insufficient cutting power and no kerf width was obtained. This highlights that power is a critical factor for achieving cutting penetration. The kerf widths for the unirradiated group (Experiments 2, 3, and 4) were 0.438 mm, 0.458 mm, and 0.477 mm, respectively, whereas those for the irradiated group (Experiments 6, 7, 8, 9, and 10) were 0.632 mm, 0.635 mm, 0.651 mm, 0.639 mm, and 0.652 mm, respectively. Overall, the kerf widths in the irradiated group were significantly larger than those in the unirradiated group, suggesting that irradiation may have affected the performance of the optical fiber and laser cutting head, resulting in increased kerf widths.

At lower power levels, the cutting power was insufficient to fully penetrate the hexagonal tube (as observed in Experiments 1 and 5). As the power increased (e.g., Experiments 2, 3, 4, and Experiments 6, 7, 9, 10), the kerf width also increased. This indicates that higher laser power provides greater thermal input, leading to wider kerfs. A comparison between Experiments 7 and 8 (same power, different cutting speeds) revealed that a lower cutting speed (Experiment 8, 3.5 m/min) resulted in a wider kerf of 0.651 mm, while a higher cutting speed (Experiment 7, 4.5 m/min) produced a narrower kerf of 0.635 mm. From these two datasets, it can be guessed that this may be due to the longer laser interaction time at slower speeds, allowing greater heat accumulation and more extensive material melting, thereby increasing the kerf width. The exact reason needs to be obtained from subsequent and more extensive experimental data.

**Figure 12:**
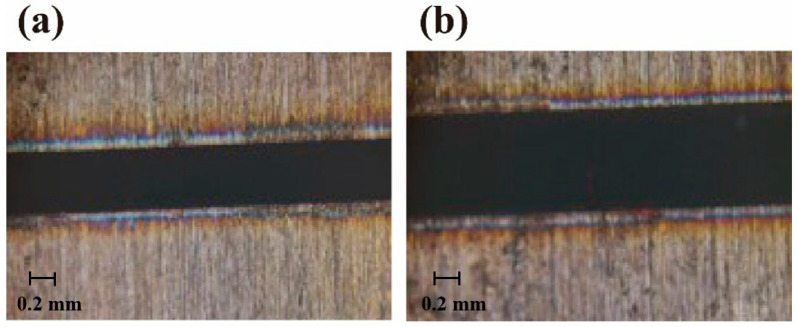
Close-up images of kerfs: (**a**) Experiment 3, (**b**) Experiment 6.

Figure 12 illustrates the laser cut kerf for the irradiated and non-irradiated experimental groups for the same parameters. It can be clearly seen that the width of the kerf in Figure 12b (0.632 mm) is wider (0.458 mm) relative to Figure 12a. The substantial increase in kerf width in the irradiated group may be attributed to changes in laser transmission efficiency or thermal energy distribution caused by irradiation. Irradiation likely induces microstructural changes in the optical fiber material, leading to more dispersed laser energy and, consequently, wider kerfs.

**Figure 13:**
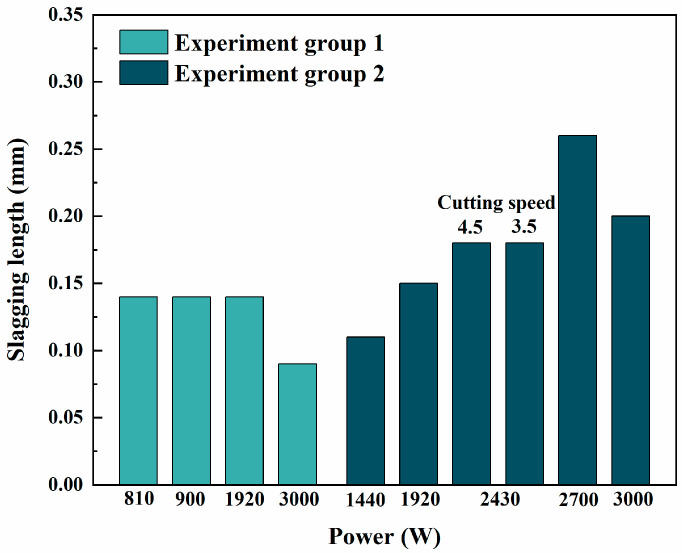
Length of spatter under different cutting parameters.

Figure 13 shows the slag length under different laser cutting parameters, comparing results from the unirradiated group (Experiments 1, 2, 3, and 4) and the irradiated group (Experiments 5, 6, 7, 8, 9, and 10). For the unirradiated group, the slag lengths were 0.14 mm, 0.14 mm, 0.14 mm, and 0.09 mm, respectively. The data indicate that increasing power (from Experiment 1 to 4) did not significantly affect slag length. In Experiment 4, slag length decreased slightly, likely due to optimized power and improved cutting efficiency. For the irradiated group, the slag lengths were 0.11 mm, 0.15 mm, 0.18 mm, 0.18 mm, 0.26 mm, and 0.20 mm, respectively. Compared to the unirradiated group, the irradiated group generally exhibited longer slag lengths, with the longest slag length of 0.26 mm recorded in Experiment 9. This suggests that irradiation affects slag generation and adhesion during the cutting process.

The data also highlight that cutting speed has a more pronounced effect on slag length than power. At slower cutting speeds (e.g., Experiment 8, with a speed of 3.5 m/min), slag length increased significantly. This can be attributed to prolonged laser interaction with the hexagonal tube surface at lower speeds, causing greater heat accumulation, which generates more molten slag and increases adhesion to the cut surface. In contrast, experiments with higher cutting speeds (e.g., Experiments 7, 9, and 10, with a speed of 4.5 m/min) showed shorter slag lengths. A comparison of Experiments 1, 2, 3, and Experiments 7, 8, 9, and 10 reveals that power has a relatively minor impact on slag length. Increasing power within a certain range (e.g., from Experiment 1 to 3 or Experiment 7 to 9) resulted in only slight variations in slag length, indicating that power is not the primary driver of slag length changes.

**Figure 14:**
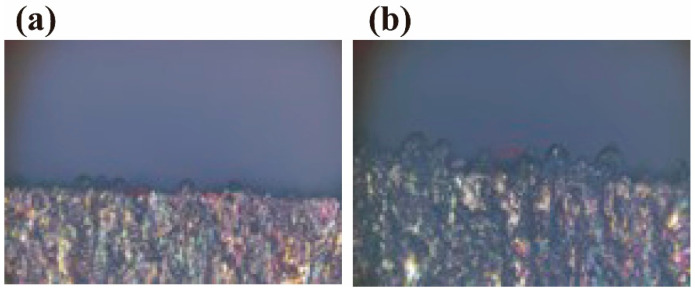
Enlarged views of the cross-section: (**a**) Experiment 3, (**b**) Experiment 7. (40× magnification.)

Figure 14 shows an enlarged cross-section of the laser cut sections of Experiment 3 and Experiment 7, and it can be observed that the amount of slag in the irradiated experimental group (0.14 mm) is more than that in the non-irradiated experimental group (0.18 mm). The cut section of the non-irradiated experimental group is relatively smoother and less rough. This may be due to irradiation-induced changes in the performance of the optical fiber and laser cutting head, which could alter laser energy transmission and focusing characteristics, thereby increasing slag generation and adhesion during the cutting process.

**Figure 15:**
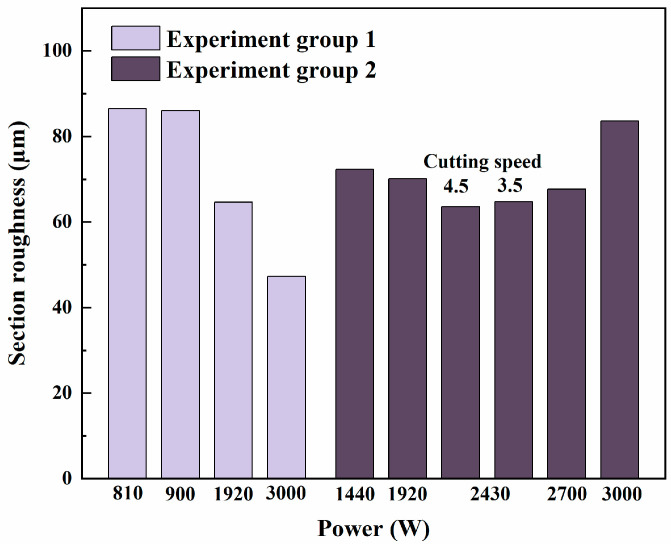
Surface roughness under different cutting parameters.

Figure 15 presents the surface roughness results for all experimental groups. Among the experiments, Experiment 4 achieved the lowest surface roughness (47.25 µm), while Experiment 1 recorded the highest (86.53 µm). These findings highlight that optimizing laser cutting parameters, such as power and cutting speed, can significantly enhance the quality of the cutting surface. A comparison of the two experimental groups shows that the impact of irradiation on surface roughness is less pronounced than its effects on kerf width (Figure 11) and slag length (Figure 13). Under low-power conditions (power < 2000 W), the irradiated group (Experiment Group 2) demonstrated even better surface roughness than the unirradiated group (Experiment Group 1). This suggests that within an appropriate power range, irradiated optical fibers can still deliver stable laser energy and maintain high cutting quality.

However, when applying the same cutting parameters as Experiment 3 (Experiment 6, power of 1920 W and cutting speed of 4.5 m/min), the irradiated group did not achieve optimal cutting quality. Subsequent experiments increased the cutting power (Experiment 7, power of 2430 W), which produced more favorable results. Comparing Experiments 8 (cutting speed of 3.5 m/min) and 7 (cutting speed of 4.5 m/min), it was observed that a cutting speed of 4.5 m/min resulted in lower surface roughness. Therefore, the optimal cutting parameters for the irradiated group were determined to be those used in Experiment 7 (power of 2430 W and cutting speed of 4.5 m/min). At the maximum power of 3000 W, the irradiated group (Experiment Group 2) successfully cut the hexagonal tube, with the surface roughness and other quality parameters remaining within acceptable limits for industrial applications. These results demonstrate that optical fibers, although damaged under the cumulative γ-irradiation dose of 10^5^ Gy, generally maintain sufficient energy transfer capability to reliably cut the hexagonal tubes of fast reactor assemblies, meeting the requirements for practical use, and retaining the potential to be applied in reprocessing power plants in the future.

## 4. Conclusions

This study systematically evaluated the performance changes in selected optical fibers under a γ-irradiation dose rate of 2000 Gy/h and a total dose of 10^5^ Gy, as well as the feasibility of laser cutting systems for use in highly irradiated environments. Through microscopic observation of the irradiated fibers, output power and temperature variation tests, and practical cutting experiments on a rotary cutting platform with an irradiated laser cutting head attached, the following conclusions were drawn:

(1) Compared to unirradiated fibers, the energy transmission capacity of these irradiated fibers showed a slight decline. When low-power transmission errors were disregarded, the maximum power loss was only 0.36%, indicating that irradiation has a minimal impact on the overall transmission efficiency. Additionally, while the irradiated fibers exhibited a temperature increase during energy transmission, their surface temperature remained below 50 °C even at an input power of 3000 W, demonstrating sufficient thermal stability for practical cutting experiments.
(2) The study successfully conducted cutting experiments on the hexagonal tubes of the fast reactor simulation assembly using irradiated optical fibers combined with an irradiated laser cutting head. Under various power and cutting speed conditions, the key cutting quality indicators, including kerf width, slag length, and surface roughness, were analyzed. The results showed that both power and cutting speed significantly influence cutting quality. The optimal cutting parameters for the irradiated group were determined to be a power of 2430 W and a cutting speed of 4.5 m/min. Furthermore, the irradiated fibers and cutting head fully met the requirements for cutting in an industrial environment.
(3) This study demonstrated that the optical components and laser cutting processes used in this experiment are feasible and stable for shearing the hexagonal tubes of a fast reactor simulation assembly under a cumulative γ-irradiation dose of 10^5^ Gy. These findings validate the applicability of this technology in high-radiation environments and provide critical technical support for the practical implementation of laser cutting technology in nuclear fuel reprocessing.

## Figures and Tables

**Table 1:** Laser cutting experiment parameters.

Experiment Group	Experiment Numbers	Power (W)	Cutting Speed (m/min)
1	1	810	4.5
	2	900	4.5
	3	1920	4.5
	4	3000	4.5
2	5	1440	4.5
	6	1920	4.5
	7	2430	4.5
	8	2430	3.5
	9	2700	4.5
	10	3000	4.5

## Data Availability

Data are contained within the article.
